# Antibodies to Henipavirus or Henipa-Like Viruses in Domestic Pigs in Ghana, West Africa

**DOI:** 10.1371/journal.pone.0025256

**Published:** 2011-09-22

**Authors:** David T. S. Hayman, Lin-Fa Wang, Jennifer Barr, Kate S. Baker, Richard Suu-Ire, Christopher C. Broder, Andrew A. Cunningham, James L. N. Wood

**Affiliations:** 1 Cambridge Infectious Diseases Consortium, University of Cambridge, Cambridge, United Kingdom; 2 Institute of Zoology, Zoological Society of London, London, United Kingdom; 3 Animal Health and Veterinary Laboratories Agency, Weybridge, United Kingdom; 4 Colorado State University, Fort Collins, Colorado, United States of America; 5 CSIRO Livestock Industries, Australian Animal Health Laboratory, Geelong, Australia; 6 Wildlife Division of the Ghana Forestry Commission, Accra, Ghana; 7 Department of Microbiology and Immunology, Uniformed Services University, Bethesda, Maryland, United States of America; Harvard Medical School, United States of America

## Abstract

Henipaviruses, Hendra virus (HeV) and Nipah virus (NiV), have Pteropid bats as their known natural reservoirs. Antibodies against henipaviruses have been found in *Eidolon helvum,* an old world fruit bat species, and henipavirus-like nucleic acid has been detected in faecal samples from *E. helvum* in Ghana. The initial outbreak of NiV in Malaysia led to over 265 human encephalitis cases, including 105 deaths, with infected pigs acting as amplifier hosts for NiV during the outbreak. We detected non-neutralizing antibodies against viruses of the genus *Henipavirus* in approximately 5% of pig sera (N = 97) tested in Ghana, but not in a small sample of other domestic species sampled under a *E. helvum* roost. Although we did not detect neutralizing antibody, our results suggest prior exposure of the Ghana pig population to henipavirus(es). Because a wide diversity of henipavirus-like nucleic acid sequences have been found in Ghanaian *E. helvum*, we hypothesise that these pigs might have been infected by henipavirus(es) sufficiently divergent enough from HeVor NiV to produce cross-reactive, but not cross-neutralizing antibodies to HeV or NiV.

## Introduction

The genus *Henipavirus* in the family *Paramyxoviridae* is comprised of Hendra (HeV) and Nipah (NiV) viruses. These viruses use bats of the family *Pteropodidae* as their natural reservoir hosts [1], [2]. Henipaviruses have a remarkably wide susceptible host range and represent some of the most pathogenic viruses known, each capable of causing an often fatal encephalitis or severe respiratory disease and both are classified as biosafety level 4 pathogens. Outbreaks of NiV in Malaysia, India and Bangladesh have had case fatality rates ranging from 40–90% [3], [4], [5]. The Malaysian NiV epidemic led to over 265 human encephalitis cases, with 105 deaths [4]. To date, two domestic species are known to have served as amplifying hosts for henipaviruses prior to transmission to humans; horses for HeV and pigs for NiV. Infected pigs acted as amplifier hosts for NiV during the Malaysian NiV outbreak, and over one million pigs were culled to contain the epidemic [6], [7]. Furthermore, both cats and dogs have been found to be positive (NiV-cats) or seropositive (NiV and HeV-dogs) [7], [8].

We previously reported serological evidence for henipavirus infection in *Eidolon helvum* bats in Ghana, West Africa [9]. *Eidolon helvum* roosts in large colonies, reaching several million in number, and has a wide distribution across the African continent. Those findings thus extended the range of henipaviruses from Asia and Australasia to Africa. A subsequent study found henipavirus-like nucleic acid in faecal samples from *E. helvum* in Ghana [10]. We therefore hypothesised that domestic animal species that have previously acted as amplifier hosts elsewhere may have been exposed to henipavirus infection in Africa.

To test this hypothesis, we screened a selection of domestic animal sera from animals within the grounds of the 37 Military Hospital, Accra, Ghana, where a large *E. helvum* colony (up to 1 million individual animals) resides for approximately 6 months during each dry season.

## Methods

Ethical approval for this project (WLE/0467) was received from the Zoological Society of London Ethics Committee and locally from the Ghanaian Veterinary Services Directorate. Serum samples were collected in June 2007 from 2 cats, 2 dogs, 10 sheep and 15 goats. In addition, 97 pig samples were available from 2 villages, collected as part of a *Trypanosoma* study undertaken at the Veterinary Services Laboratory, Ghana. Both villages are in the Suhum/Kraboa/Coalta district, Eastern Region, about 70 km north of Accra. Sample numbers P1–48 and P50 were from 25 households in Kwesikonfo (N 6°33; W 0° 33) and P52–64 and P66–100 from 11 households in Zorh (N 5°59 W 0°21). The bat-pig contact history was unknown. However, villages contain fruit trees and the pigs were housed in open pens (1–10/pen), with some running free during the day, and bats of numerous species have been caught by the authors foraging in other villages in the region [9], [11].

All sera were tested for antibodies binding to both a HeV and NiV recombinant soluble G glycoprotein (sG) using a Luminex® multiplexed binding assay, as described previously [9], [12]. Viral envelope glycoproteins have previously been demonstrated to be the primary protein for paramyxovirus attachment and virus entry, and also the principle viral antigens that inducer neutralizing antibodies in hosts [13]. The recombinant sG proteins used in the Luminex assay were generated using a mammalian expression system in a soluble and oligomeric form by removing the transmembrane domain, and purified sG was coupled to microspheres as described previously [12]. For all test samples sG_NiV_ and sG_HeV_-coupled microsphere subsets were pre-mixed and incubated with sera, followed by incubation with biotinylated Protein A/G and streptavidin–phycoerythrin. Antibodies bound to the sG_NiV_ or sG_HeV_ coated beads, which are spectrally distinct, are quantified by the fluorescence emitted by phycoerythrin. This is read as the median fluorescence intensity (M.F.I.). Gamma-irradiated positive pig and cat sera controls from naturally or experimentally infected animals, and negative controls from each species were used. Putative positive sera, with M.F.I. titres 3-fold above the negative sera M.F.I., were then tested using a Luminex ephrin-B2 receptor blocking (inhibition) assay and by virus neutralization tests (VNTs) [12]. Field samples of HeV infected horse and NiV infected pig sera has previously been demonstrated to block ephrin-B2-G glycoprotein interactions in a dose dependent fashion [12]. For the VNTs, sera were tested against both NiV and HeV at a 1∶10 dilution.

Finally, 7 pig sera with high Luminex binding titres, and 1 with a low Luminex binding titre (P17) were tested by western blot (WB). The WB was performed using purified recombinant Nipah virus nucleocapsid (N) protein produced as previously described [14]. Briefly, 50µg of purified NiV N protein was separated using SDS-PAGE on a 12% gel using a wide preparative comb, followed by electroblotting onto a nitrocellulose membrane and blocked overnight in blocking buffer (5% w/v Skim Milk Powder in TBS buffer). The nitrocellulose membrane was cut into strips and incubated for 1h with individual sera (diluted 1∶50 in blocking buffer). Negative controls were a negative pig serum and blocking buffer alone, with NiV-neutralizing human and pig sera as positive controls. Following washing, the strips were incubated for 1h with a protein A/G alkaline phosphatase conjugate (Thermo-Fisher Scientific Inc., USA) at 1∶2000 in blocking buffer. Following washing, alkaline phosphatase substrates were added and allowed to develop for 15 minutes. The marker used was Benchmark prestained protein ladder (Invitrogen, UK).

## Results

**Figure 1:**
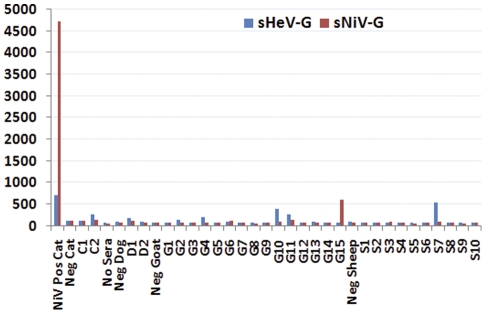
The M.F.I. of anti-NiV (Red) and HeV (Blue) antibodies from 2 cats (C), 2 dogs (D), 10 sheep (S) and 15 goats (G) from Ghana with NiV positive cat and negative (all species) control.

**Figure 2:**
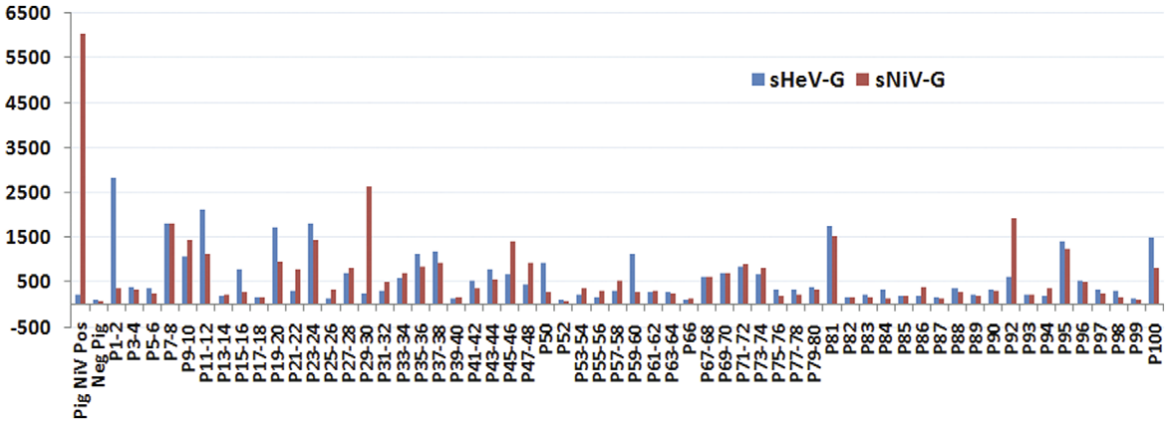
The median fluorescent intensities (M.F.I.) of 97 pig (P) sera from Ghana tested in pools of 2 or singly for antibodies against Nipah (NiV-Red) and Hendra (HeV-Blue) virus soluble glycoprotein (sG) attached to microspheres on the Luminex platform. Positive and negative serum controls are shown.

**Figure 3:**
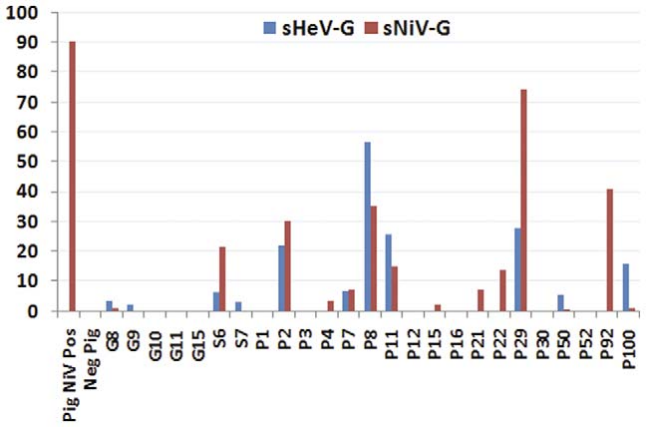
Percentage inhibition of NiV and HeV sG-Ephrin-B2 receptor by Luminex binding assay positive pig (P) sera, with NiV positive and negative controls.

One goat (G) and one sheep (S) sample each had M.F.I. titres well above 3 times background for NiV and HeV respectively. G15′s titre was 584 M.F.I. against NiV (>3 times mean negative sera M.F.I. of 62), but only produced a titre of 71.5 M.F.I. against HeV (negative sera M.F.I. 62). Sheep S7′s M.F.I. titre was 521 against HeV (>3 times mean negative sera M.F.I. of 81), but only 86 M.F.I. against NiV (negative sera M.F.I. 66) (see Figure 1). Neither these, nor 4 further goat and 1 further sheep sera tested, were positive using the inhibition assay. Pig sera were tested in 2-sample pools (N = 74 samples) or individually (N = 23) (see Figure 2). Inhibition was then tested on individual samples for those with high binding readings (see Figure 3). Of those tested, 5/18 (5.2% of the total pig sera) had high levels (>20%) of inhibition. Sample P29 gave 74% inhibition. This is high and comparable to the NiV-positive pig control inhibition of 90%. P29 was one of a pool of sera tested with another sample by Luminex sG binding, and therefore diluted 1∶2 for the original test. This pool had a titre of 2638 M.F.I., compared to a NiV positive pig titre of 6043 M.F.I.

**Figure 4:**
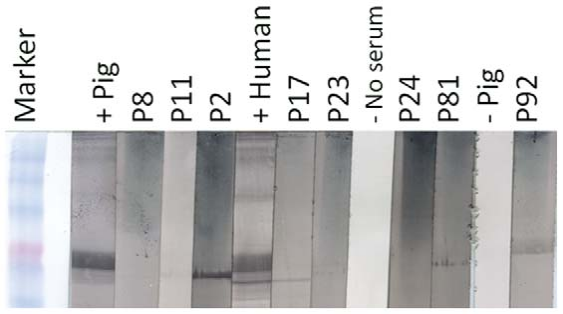
The results of western blot analysis against Nipah virus nucleocapsid protein of seven Ghanaian pig sera (designated P) with high Luminex binding assay titres and one (P17) with a low binding assay titre against soluble henipavirus glycoproteins. The marker is BenchMark Pre-stained Protein Ladder (Invitrogen); the positive sera NiV virus neutralization test positive pig and human field sera; the negative control sera negative pig and skimmed milk powder (SMP).

All sera tested for inhibition (5 goat, 2 sheep and 18 pig) were tested by VNTs, but no sera tested positive using VNTs. However, one (P92) of the seven binding-antibody positive pig sera tested positive by WB against the N protein (Figure 4), thus confirming non-neutralizing antibody against another NiV protein. No further sample was available for P29 for testing by WB. The additional WB staining seen in all samples except P92 is thought to be non-specific background staining.

## Discussion

Our results suggest evidence of prior exposure of the Ghana pig population to henipavirus(es). The wide diversity of henipavirus-like nucleic acid sequences that have been found in Ghanaian bats [10] means that it is possible that these pigs have been infected by henipavirus(es) divergent enough from HeV or NiV to produce glycoprotein binding antibodies, but not HeV or NiV neutralizing antibodies [15].

Luminex binding assay has been demonstrated to give preferentially higher M.F.I. for the virus that induced antibody from field sera of NiV infected pigs from the 1998–1999 NiV outbreak in Malaysia and Singapore, and HeV infected horses from the 1994 HeV outbreak [12]. While it is possible that the positive results found against both virus sG in this study are due to broad high background binding, the lack of this finding in Malaysian pig sera from the 1998–1999 outbreak and the relatively high inhibition of ephrin-B2-G glycoprotein interactions (Figure 3) are suggestive of previous exposure to henipaviruses. In addition to this, binding was confirmed on another platform, WB, and against another protein (N) by a single serum sample. This sample (P92) had demonstrated 40% sG-Ephrin B2 receptor inhibition and had a Luminex binding M.F.I. titre 27-fold higher than the negative pig control serum. Although the non-specific staining by other positive sera is undesirable, pig sera have been shown previously to be reactive for non-specific stains, particularly to *E. coli* proteins. However, given binding antibody had previously been demonstrated against sG NiV proteins in this sample of pig sera, we believe this adds further evidence of infection by a NiV or Nipah-related virus in these populations.

The mechanisms of virus neutralization are complex and could involve more antigenic sites than those required for simple receptor binding or inhibition. The failure of finding VNT positive sera therefore may be due to the divergent nature of the viruses inducing antibody. As has been shown in studies on bats in Ghana, only a relatively small proportion of those seropositive by Luminex assay were VNT positive, and subsequent studies by others have shown related but divergent henipavirus sequences from the same bat species. Furthermore, previous studies have shown the NiV antibody positive pig sera produced lower titres than human (NiV, HeV), bat (HeV) and horse (HeV) positive sera using all three (binding, receptor inhibition and neutralization) assays [12].

Laboratory studies suggest viral replication in bats is limited [16], [17] and bat-to-human transmission outside of Bangladesh is yet to be reported. Therefore, evidence of infection in potential amplifying hosts in Ghana is an important finding, whether due to multiple introductions or a single introduction of infection with subsequent pig-to-pig transmission. It is unknown which is the cause of the Ghanaian pig serological results here, however, both are important events that may lead to henipavirus emergence by altering infection dynamics within the populations on subsequent re-introduction [18]. Further sera from these animals are unavailable for additional testing; however, future sampling should be age-specific in order to make inferences relating to infection dynamics in the pig populations.

Finally, *E. helvum* frequently roosts in urban areas, is a source of bushmeat [9], [11] and is known to forage in semi-urban areas [19], and we are therefore currently increasing the study size and extending it to include humans and animals in high risk groups of exposure to bats in order to determine the likelihood of exposure to potentially-fatal zoonotic viruses.
